# The Impact of Avatar Appearance on the Persuasiveness of a Short Video Encouraging Physical Activity: A Randomized Observational Study

**DOI:** 10.7759/cureus.78582

**Published:** 2025-02-05

**Authors:** Momoko Tohyama, Ryo Momosaki, Kazuma Tora, Tsuyoshi Okuhara

**Affiliations:** 1 Department of Rehabilitation Medicine, Mie University Graduate School of Medicine, Tsu, JPN; 2 Department of Rehabilitation, Akiyama Clinic, Takamatsu, JPN; 3 Department of Health Communication, School of Public Health, Graduate School of Medicine, The University of Tokyo, Tokyo, JPN

**Keywords:** acceptability, avatar, health communication, persuasiveness, self-efficacy

## Abstract

Background

Avatars have been used as tools for communication in various fields. However, the characteristics of avatars useful for health communication remain unclear. This study aimed to examine the impact of medical avatars (MAs) on health communication.

Methodology

An anonymous questionnaire survey was conducted among individuals aged 18-59 years. Participants were randomly assigned to view one of four avatar-based videos (medical male, nonmedical male, medical female, or nonmedical female avatar). In this study, MAs were defined as those wearing white coats, while nonmedical avatars (NAs) wore hoodies. Total scores for perceived persuasiveness of the video, avatar acceptability, and exercise self-efficacy were then compared between the MA and NA groups.

Results

Overall, 309 participants were included in the analysis, with 160 (51.8%) classified into the MA group and 149 (48.2%) into the NA group. The MA group had higher scores for persuasiveness and acceptability than the NA group. In the subgroup analysis based on avatar experience among individuals with avatar experience, the MA group had higher persuasiveness and acceptability scores than the NA group; however, no significant difference was observed among individuals without avatar experience.

Conclusions

In health communication, avatar appearance may impact the persuasiveness and acceptability of the videos. The results of this study indicate that avatars wearing white coats were perceived as more persuasive and acceptable by the participants.

## Introduction

Technological advancements have shifted lifestyles toward increased sedentary behavior, resulting in various health issues associated with reduced physical activity [1]. Previous studies have identified sedentary behavior and physical activity levels as independent risk factors for health and reported associations with all-cause mortality and incidence of noncommunicable diseases [2,3]. In particular, individuals with long screen time tend to have longer sedentary behavior, and a study indicated a link between sedentary time and depression [4]. Thus, measures aimed at reducing sedentary time and promoting physical activity are essential. Recently, digital technologies have gained attention as novel tools to enhance physical activity for their accessibility and potential for personalized interventions [5,6]. Educational interventions with digital technology may mitigate health risks associated with a sedentary lifestyle and promote sustainable health behaviors among a broader population.

In educational interventions, avatars are used to create digital content [7]. An avatar is a virtual object used to represent a physical entity, such as a human or an animal, which can perform relatively complex actions, including facial expressions and physical reactions [8]. Given their high level of psychological safety, avatars are increasingly used as a communication tool [9], a study examined the optimal characteristics of avatars for communication [10]. In addition, avatars are applied as marketing tools in customer service, guidance, and other business sectors [11]. Although avatar-based communication generally enhances efficiency and has a strong influence on younger generations, concerns about reliability and technical errors remain significant [11]. Ensuring the reliability of avatar use is an essential challenge.

In the medical field, the use of avatars has been explored for educational and counseling purposes. For instance, the efficacy of avatars in training and evaluating communication skills was investigated among healthcare professionals [12,13]. Previous studies have shown that avatar-based interventions are useful in the treatment of patients with mental illnesses and in education and health management for patients with chronic diseases [14-16]. These findings propose that avatar-based communication can foster a sense of security and enhance the self-efficacy, adherence, and knowledge of patients. Furthermore, in avatar-assisted counseling, the appearance of the avatar and the counseling situation have been shown to influence participants’ willingness to engage in counseling [17]. Thus, the use of avatars in healthcare for treatment and education has shown significant progress, and avatar-based health communication is expected to develop further.

However, the characteristics of avatars that are most useful for health communication remain underexplored. Specifically, which avatar features enhance communication persuasiveness to encourage healthy behavior is unclear. Previous studies have examined the association between the clothing of doctors or physical therapists and trustworthiness perceived by patients. For instance, a Japanese study assessed patient preferences and trust in various styles of doctor clothing (semiformal, white coats, surgical scrubs, and casual wear). Many patients chose white coats as the most preferred clothing for both male and female doctors, indicating that the clothing could affect the trust in medical professionals [18]. Rufa'i et al. examined the relationship between the clothing of physiotherapists and patients’ trust and comfort. The results showed that 89% of the participants reported higher trust in physiotherapists wearing white coats [19]. Therefore, wearing professional clothing, such as a white coat, is considered crucial in enhancing trustworthiness during medical or health-related communication. These findings indicate the potential applicability of video-based health communication using avatars, with the expectation that avatars resembling medical professionals could have similar positive impacts. The identification of the characteristics of avatars that are highly persuasive in health communication may contribute to the implementation of higher-quality health communication.

This study aimed to investigate the characteristics of avatars that enhance the quality of health communication through a questionnaire-based survey and hypothesized that using an avatar with a medical professional appearance could improve the quality of health communication. Participants watched videos featuring avatars, and perceived persuasiveness, acceptability, and exercise self-efficacy were assessed.

## Materials and methods

Study design

This randomized observational study of Japanese participants examined the impact of medical avatars (MAs) on health communication. After providing consent, the participants viewed a video featuring an avatar and completed a questionnaire via Google Forms. Informed consent was obtained through an explanatory document attached to the Google Forms. This study was approved by the Ethics Committee of Mie University Hospital (H2024-136). As this is an observational study, clinical trial registration was not required.

Participants

This study included individuals aged 18-59 years who consented to participate in the study. Individuals unable to use the video-viewing devices were excluded from participation. Participants were recruited through posts on an account with approximately 1,800 followers on platform X. The survey was conducted from September 5 to October 6, 2024.

Sample size

The sample size was calculated using G*Power 3.1.9.7 (Heinrich Heine University, Düsseldorf, Germany), with a primary outcome comparison between the two groups using a t-test, effect size of 0.3, significance level of 5%, and power of 80%. According to Cohen’s guidelines, an effect size of 0.3 is considered small to medium [20]. This calculation resulted in an approximate sample size of 276 participants [21]. The target sample size was 300 participants aged 18-59 years.

Intervention content

**Figure 1:**
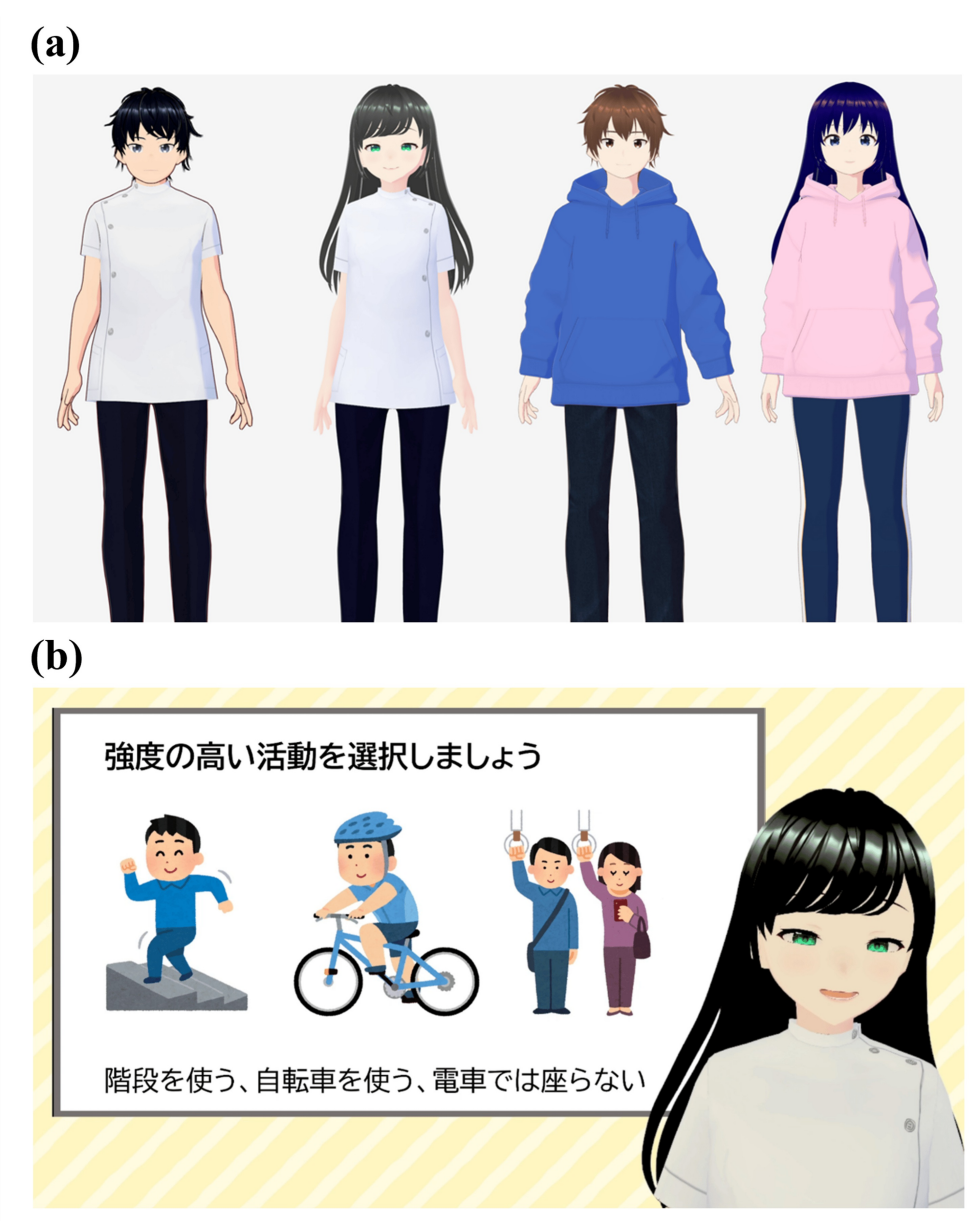
Visual representation of the videos. (a) Avatars used in the videos. (b) Screenshot of the video content as viewed, showing the text: “Choose high-intensity activities. For instance, take the stairs, ride a bicycle, and avoid sitting on the train." Screenshot of the video taken by co-author Ryo Momosaki.

Four types of avatars were created: male MA, female MA, male nonmedical avatar (NA), and female NA. To account for potential differences in how male and female participants might perceive avatars of the same or opposite sex, both male and female avatars were created for each avatar type (MA and NA). This design was implemented to control for any underlying sex-related biases or preferences in participants’ responses. The MA wore white coats, whereas the NA wore casual hoodies, with no other differences aside from appearance and gendered voices (Figure 1).

Participants were randomly assigned to view one of these avatars. The assignment was conducted using the shuffle feature of Google Forms, which randomized the order in which the videos were displayed, although the specific algorithm behind the shuffle is not publicly disclosed. Participants were instructed to watch only the video that appeared at the top of the randomized list and report the corresponding video number. This method ensured random group allocation, minimizing allocation bias and maintaining equality across groups.

The videos were approximately 2 minutes long and highlighted the importance of exercise while introducing practical ways to increase physical activity levels. Participants were not provided with specific instructions regarding viewing conditions such as viewing time or device type.

Outcomes

The primary outcome was the total score for the perceived persuasiveness of the video to evaluate the overall impact of the avatar. Perceived persuasiveness was measured using the validated questionnaire of Thomas et al., comprising nine items across three factors: effectiveness, quality, and capability, rated on a 7-point scale from *strongly disagree* to *strongly agree* [22]. The secondary outcomes were the total scores for each of the following: acceptability of the avatar and exercise self-efficacy. Acceptability of the avatar was assessed using a 10-point scale questionnaire based on five items (knowledge, trust, care, approachability, and comfort), adapted from a systematic review investigating the impact of physicians’ clothing on patient preference and satisfaction [23]. In this study, a questionnaire on the acceptability of avatars was developed based on the study by Petrilli et al. [24]. Exercise self-efficacy was measured using a validated scale by Oka, assessing confidence in exercising under five conditions that could hinder physical activity: physical fatigue, mental stress, lack of time, non-routine life, and bad weather [25]. Responses were recorded on a 5-point scale from *not at all confident* to *very confident*. Scores were summed across all items, except for the non-routine life condition, which was an irrelevant item. All outcomes were measured immediately after viewing the video.

The extracted background information included age, gender, educational level, healthcare student or professional status, and experience with avatars. Avatar experience was defined based on whether the individual regularly watched avatar-based streams or videos, such as VTuber content, or frequently used avatars in games.

Statistical analysis

Participants were classified into the MA and NA groups based on the avatar type viewed, and the background characteristics and outcomes were compared. Because this study aimed to evaluate the impact of MAs on health communication, a gender-based analysis of the avatars was not performed. Categorical data were presented as absolute numbers and percentages and compared between the groups using the χ² test. Continuous data were presented as means ± standard deviations and compared using t-tests. Subgroup analysis based on avatar experience was also performed. Statistical analysis was conducted using SPSS version 29.0 (IBM Corp., Armonk, NY), with significance defined as two-sided (*P* < 0.05).

## Results

**Figure 2:**
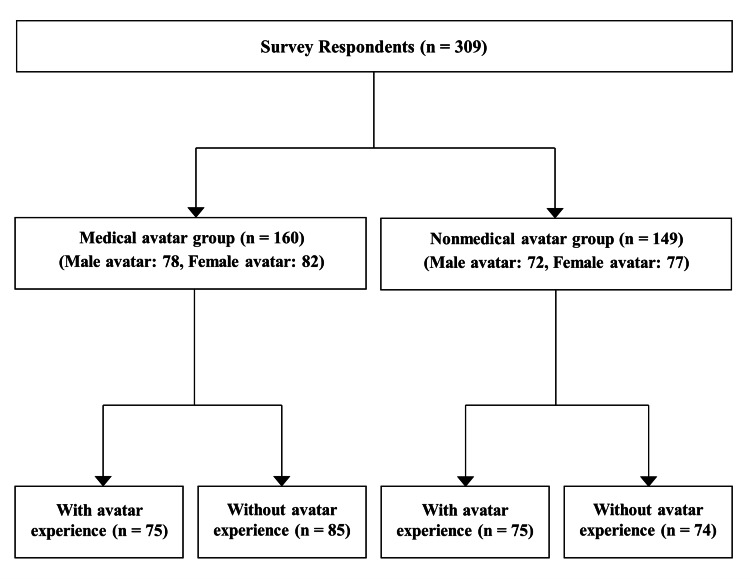
Overview of the study design.

In total, 309 participants completed the survey, with 160 viewing a video for MA and 149 viewing a video for NA (Figure 2). Participant characteristics are presented in Table 1. No significant differences were observed in gender, age, or other background characteristics between the MA and NA groups.

**Table 1:** Baseline characteristics of the participants (n = 309). Note: Categorical data were analyzed using χ^2^ tests, and continuous data were analyzed using t-tests. Significance was set at *P* < 0.05.

Characteristics	Medical avatar group (*n* = 160)	Nonmedical avatar group (*n* = 149)	*P*-value
Age (years), *n* (%)			0.428
18-29	50 (31.3)	53 (35.6)	
30-39	60 (37.5)	43 (28.9)	
40-49	31 (19.4)	35 (23.5)	
50-59	19 (11.9)	18 (12.1)	
Gender, *n* (%)			0.510
Male	90 (56.3)	74 (49.7)	
Female	68 (42.5)	73 (49.0)	
Other	2 (1.3)	2 (1.3)	
Healthcare students/professionals, *n* (%)	107 (66.9)	92 (61.7)	0.347
Educational levels, *n* (%)			0.793
<9 years	2 (1.3)	1 (0.7)	
10-12 years	21 (13.1)	24 (16.1)	
>12 years	135 (84.4)	123 (82.6)	
Other	2 (1.3)	1 (0.7)	
Experience with avatars, *n* (%)	75 (46.9)	75 (50.3)	0.543

**Figure 3:**
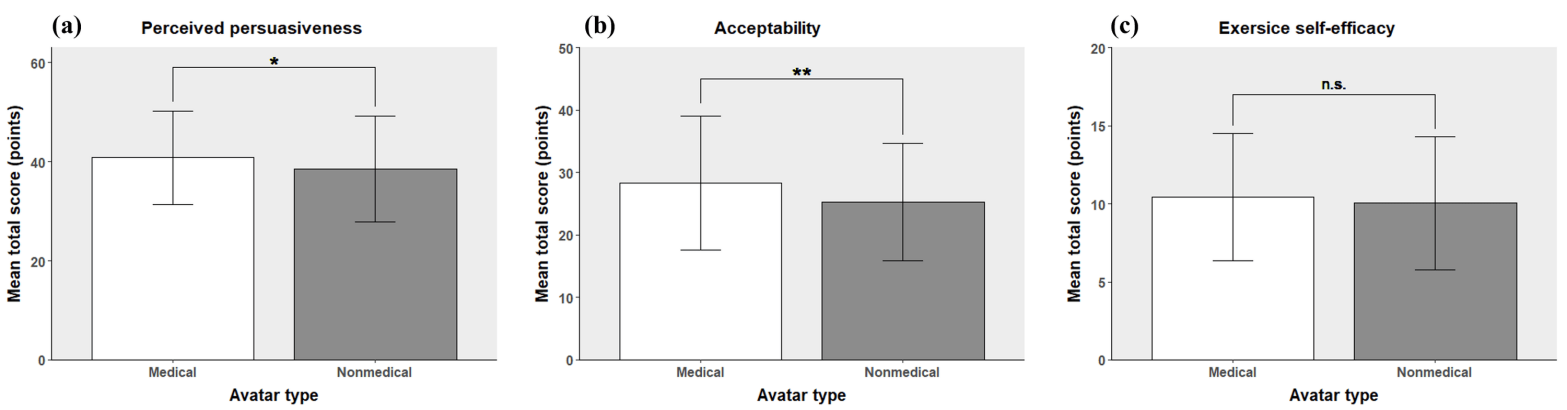
Main results of the analysis. (a) Mean total score for perceived persuasiveness, (b) mean total score for acceptability, and (c) mean total score for exercise self-efficacy. Note: The significance of the results was determined using t-tests, with significance defined as *P* < 0.05. Bars represent the mean total scores for each measure, and error bars indicate ± standard deviation (SD). Significance is indicated as follows: *P < 0.05. **P < 0.01. ***P < 0.001. Results with n.s. indicate no significant difference. The score ranges for each outcome were as follows: perceived persuasiveness (9-63), acceptability (5-50), and exercise self-efficacy (4-20).

Outcome comparisons between the groups are shown in Figure 3. The MA group had significantly higher scores for avatar perceived persuasiveness (40.79 ± 9.39 vs. 38.48 ± 10.65, *P* = 0.044) and acceptability (28.29 ± 10.67 vs. 25.30 ± 9.38, *P* = 0.009) than the NA group. No significant difference was found in exercise self-efficacy between the two groups (10.43 ± 4.09 vs. 10.04 ± 4.25, *P* = 0.419). The comparative results for all items are provided in Appendix A.

**Figure 4:**
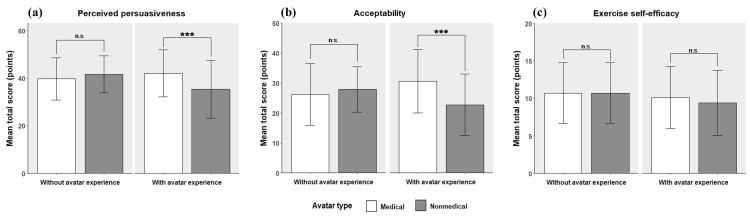
Results of the subgroup analysis. (a) Mean total score for perceived persuasiveness, (b) mean total score for acceptability, and (c) mean total score for exercise self-efficacy. Note: The significance of the results was determined using t-tests, with significance defined as *P* < 0.05. Bars represent the mean total scores for each measure, and error bars indicate ± standard deviation (SD). Significance is indicated as follows: *P < 0.05. **P < 0.01. ***P < 0.001. Results with n.s. indicate no significant difference. The score ranges for each outcome were as follows: perceived persuasiveness (9-63), acceptability (5-50), and exercise self-efficacy (4-20).

The subgroup analysis based on avatar experience is shown in Figure 4. Among participants with avatar experience, the MA group scored significantly higher on perceived persuasiveness (42.03 ± 9.89 vs. 35.40 ± 12.19, *P* < 0.001) and acceptability (30.67 ± 10.58 vs. 22.76 ± 10.29, *P* < 0.001) than the NA group. In contrast, no significant differences were observed in perceived persuasiveness (39.71 ± 8.84 vs. 41.59 ± 7.75, *P* = 0.153), acceptability (26.20 ± 10.37 vs. 27.88 ± 7.60, *P* = 0.243), and exercise self-efficacy (10.69 ± 4.04 vs. 10.68 ± 4.08, *P* = 0.977) among participants without avatar experience. The comparative results for all items are provided in Appendix B.

## Discussion

In this study, a questionnaire survey was conducted to examine the impact of MAs on health communication. The results indicated that avatars wearing white coats received higher ratings for perceived persuasiveness and acceptability than avatars wearing hoodies.

The MA group rated the persuasiveness and acceptability of the videos higher than the NA group; thus, these outcomes may be impacted by avatar appearance. In face-to-face communication, studies have shown that healthcare providers wearing professional clothing can enhance patients’ trust and sense of security [18,19,24,26]. Similarly, avatars dressed in professional clothing may increase perceived persuasiveness and acceptability in video-based health communication.

Despite similarities between the results of the previous study and the present study, the effect sizes were larger in the former. Specifically, in the previous study, the mean acceptability scores for doctors wearing white coats were all above 8 [24], whereas in the present study, the mean acceptability scores for avatars wearing white coats remained in the 5-point range. This difference may be explained by variations in the target population and the attributes of the speakers. The previous study focused on patients, whereas the present study included individuals aged 18-59. Patients may have been more strongly influenced by the acceptability of a physician’s clothing. Another factor to consider is the difference in speaker attributes. Although the previous study examined the acceptability of individuals explicitly identified as “doctors,” the present study did not specify the identity of the avatar. As a result, the lack of clarity regarding the title and expertise of the avatar may have reduced acceptability. These differences in target population and speaker attributes may have contributed to the differences in effect sizes.

The effect of avatar appearance on exercise self-efficacy was also investigated. However, no significant difference in self-efficacy was found between the MA and NA groups. This may reflect differences in participants’ stages of behavior change as per the transtheoretical model [27], indicating that the content may not have been optimal for enhancing exercise self-efficacy for all participants. Furthermore, the avatar video used in this study was only approximately 2 minutes long, which may not have been enough to positively impact self-efficacy. Thus, identifying avatar characteristics that are useful in improving self-efficacy will require intervention studies with adequately timed, personalized video content.

A subgroup analysis based on avatar experience was conducted. Among participants with avatar experience, the MA group rated the perceived persuasiveness and avatar acceptability higher than the NA group. However, among participants without avatar experience, no difference was found between the two avatar types across any of the items. This indicates that among individuals with avatar experience, the appearance of avatars may influence the trustworthiness of the video and acceptability, whereas individuals without avatar experience are less impacted by the appearance of the avatar. Although this is currently a hypothesis, the results may be due to the higher attention to avatars among experienced users, making them more receptive to the avatar appearance. Future studies should investigate the association between avatar experience and attention to avatars.

To the best of our knowledge, this is the first study that examined the impact of MAs on health communication. Four types of avatar videos were used in this study: male MA, female MA, male NA and female NA. The inclusion of both male and female avatars enabled us to control for the impacts of avatar gender, which is often cited as a limitation in avatar and YouTuber research [28], thus allowing a clearer comparison of the impact of avatar appearance.

However, this study has several limitations. First, the generalizability of the results is limited because participants were recruited through social media, excluding those who do not use such platforms. Second, our study focused on health communication, which may have attracted individuals with a particular interest in the topic, potentially introducing selection bias. Third, environmental factors such as viewing time, location, and device were not controlled, which might have influenced participants’ psychological responses. Finally, this study evaluated perceived persuasiveness and acceptability immediately after video viewing and therefore could not assess long-term impacts such as behavior change.

## Conclusions

In this study, avatar appearance was found to influence the perceived trustworthiness and acceptability of videos in health communication. Avatars wearing white coats were associated with higher trustworthiness and acceptability. These findings indicate that tailoring avatar appearance may enhance the quality of health communication.

Future research should investigate the long-term effects of avatar appearance on behavior change and health outcomes. In addition, examining how not only avatar attire but also other customization factors, such as voice, personality traits, and speaking style-affect the effectiveness of avatar-based interventions is important. Identifying optimal methods for video-based health communication may improve patient education and contribute to better health outcomes on a population level.

## Appendices

Appendix A 

**Table 2:** Results for all survey items (n = 309) Note. The significance of the results was determined using t-tests, with significance defined as p < 0.05.

Measure Outcomes (mean ± SD)	Medical avatar group (n = 160)	Nonmedical avatar group (n = 149)	p-value
This video will cause changes in my behavior.	4.22 ± 1.21	3.93 ± 1.46	0.058
This video causes me to make some changes in my behavior.	4.21 ± 1.31	3.89 ± 1.48	0.046
After viewing this video, I will make changes in my attitude.	3.71 ± 1.35	3.50 ± 1.42	0.173
This video is accurate.	5.11 ± 1.25	4.95 ± 1.47	0.307
This video is trustworthy.	4.81 ± 1.35	4.58 ± 1.39	0.157
I believe this video is true.	5.36 ± 1.20	5.19 ± 1.53	0.288
This video has the potential to change viewer behavior.	4.47 ± 1.44	4.17 ± 1.44	0.062
This video has the potential to influence viewer behavior.	4.59 ± 1.45	4.26 ± 1.46	0.046
This video has the potential to inspire viewers.	4.32 ± 1.44	4.02 ± 1.50	0.076
Perceived persuasiveness (total points)	40.79 ± 9.39	38.48 ± 10.65	0.044
How knowledgeable does this avatar appear?	5.70 ± 2.13	4.86 ± 2.08	0.001
How trustworthy does this avatar appear?	5.55 ± 2.22	4.77 ± 2.09	0.002
How caring does this avatar appear?	5.77 ± 2.34	5.17 ± 2.16	0.020
How approachable does this avatar appear?	5.67 ± 2.48	5.36 ± 2.21	0.253
How comfortable does this avatar make you feel?	5.61 ± 2.48	5.14 ± 2.23	0.084
Acceptability (total points)	28.29 ± 10.67	25.30 ± 9.38	0.009
I have the confidence to move my body even when I am a little tired.	2.80 ± 1.17	2.63 ± 1.21	0.214
I have the confidence to move my body even when I am not in the best mood.	2.56 ± 1.17	2.49 ± 1.20	0.625
I have the confidence to move my body even when I am busy and have no time.	2.48 ± 1.18	2.36 ± 1.18	0.404
I have the confidence to move my body even during holidays or breaks. (irrelevant item)	–	–	–
I have the confidence to move my body even when the weather is not very good.	2.59 ± 1.18	2.56 ± 1.17	0.785
Exercise self-efficacy (total points)	10.43 ± 4.09	10.04 ± 4.25	0.419

Appendix B 

**Table 3:** Subgroup analysis results for all survey items (n = 309) Note. The significance of the results was determined using t-tests, with significance defined as p < 0.05.

Measure Outcomes (mean ± SD)	Medical avatar group (n = 160)	Nonmedical avatar group (n = 149)	p-value
This video will cause changes in my behavior.			
With avatar experience	4.24 ± 1.30	3.57 ± 1.58	0.006
Without avatar experience	4.21 ± 1.13	4.30 ± 1.22	0.650
This video causes me to make some changes in my behavior.			
With avatar experience	4.27 ± 1.47	3.59 ± 1.66	0.009
Without avatar experience	4.15 ± 1.16	4.19 ± 1.20	0.847
After viewing this video, I will make changes in my attitude.			
With avatar experience	3.65 ± 1.54	3.16 ± 1.61	0.058
Without avatar experience	3.76 ± 1.16	3.84 ± 1.11	0.686
This video is accurate.			
With avatar experience	5.24 ± 1.29	4.69 ± 1.75	0.032
Without avatar experience	4.99 ± 1.22	5.20 ± 1.07	0.240
This video is trustworthy.			
With avatar experience	5.13 ± 1.35	4.33 ± 1.65	0.002
Without avatar experience	4.52 ± 1.28	4.84 ± 1.02	0.083
I believe this video is true.			
With avatar experience	5.56 ± 1.17	4.89 ± 1.79	0.008
Without avatar experience	5.18 ± 1.20	5.49 ± 1.16	0.101
This video has the potential to change viewer behavior.			
With avatar experience	4.67 ± 1.61	3.71 ± 1.58	<0.001
Without avatar experience	4.31 ± 1.26	4.64 ± 1.10	0.082
This video has the potential to influence viewer behavior.			
With avatar experience	4.80 ± 1.53	3.85 ± 1.61	<0.001
Without avatar experience	4.40 ± 1.36	4.66 ± 1.16	0.193
This video has the potential to inspire viewers.			
With avatar experience	4.47 ± 1.56	3.60 ± 1.64	0.001
Without avatar experience	4.19 ± 1.32	4.45 ± 1.20	0.201
Perceived persuasiveness (total points)			
With avatar experience	42.03 ± 9.89	35.40 ± 12.19	<0.001
Without avatar experience	39.71 ± 8.84	41.59 ± 7.75	0.153
How knowledgeable does this avatar appear?			
With avatar experience	6.23 ± 2.07	4.20 ± 2.16	<0.001
Without avatar experience	5.24 ± 2.09	5.53 ± 1.78	0.345
How trustworthy does this avatar appear?			
With avatar experience	5.93 ± 2.28	4.23 ± 2.26	<0.001
Without avatar experience	5.21 ± 2.12	5.32 ±1.76	0.716
How caring does this avatar appear?			
With avatar experience	6.21 ± 2.47	4.68 ± 2.38	<0.001
Without avatar experience	5.38 ± 2.14	5.66 ± 1.78	0.361
How approachable does this avatar appear?			
With avatar experience	6.21 ± 2.46	4.87 ± 2.49	0.001
Without avatar experience	5.19 ± 2.40	5.86 ± 1.77	0.044
How comfortable does this avatar make you feel?			
With avatar experience	6.08 ± 2.53	4.79 ± 2.50	0.002
Without avatar experience	5.19 ± 2.37	5.50 ± 1.88	0.359
Acceptability (total points)			
With avatar experience	30.67 ± 10.58	22.76 ± 10.29	<0.001
Without avatar experience	26.20 ± 10.37	27.88 ± 7.60	0.243
I have the confidence to move my body even when I am a little tired.			
With avatar experience	2.75 ± 1.23	2.43 ± 1.27	0.120
Without avatar experience	2.85 ± 1.11	2.84 ± 1.12	0.959
I have the confidence to move my body even when I am not in the best mood.			
With avatar experience	2.44 ± 1.16	2.32 ± 1.25	0.545
Without avatar experience	2.66 ± 1.18	2.66 ± 1.13	0.986
I have the confidence to move my body even when I am busy and have no time.			
With avatar experience	2.47 ± 1.18	2.25 ± 1.19	0.276
Without avatar experience	2.48 ± 1.19	2.47 ± 1.16	0.960
I have the confidence to move my body even during holidays or breaks. (Irrelevant item)			
With avatar experience	–	–	–
Without avatar experience	–	–	–
I have the confidence to move my body even when the weather is not very good.			
With avatar experience	2.47 ± 1.21	2.41 ± 1.18	0.786
Without avatar experience	2.71 ± 1.15	2.70 ± 1.15	0.986
Exercise self-efficacy (total points)			
With avatar experience	10.12 ± 4.15	9.41 ± 4.35	0.311
Without avatar experience	10.69 ± 4.04	10.68 ± 4.08	0.977
